# Regional Anesthesia with Spontaneous Breathing for Trans-Axillary Surgery in Thoracic Outlet Syndrome: A Retrospective Comparative Study

**DOI:** 10.3390/jcm14020601

**Published:** 2025-01-18

**Authors:** Francesco Stilo, Alessandro Strumia, Vincenzo Catanese, Nunzio Montelione, Eleonora Tomaselli, Giuseppe Pascarella, Fabio Costa, Alessandro Ciolli, Ferdinando Longo, Alessia Mattei, Lorenzo Schiavoni, Alessandro Ruggiero, Francesco Alberto Codispoti, Julia Paolini, Felice Eugenio Agrò, Francesco Spinelli, Massimiliano Carassiti, Rita Cataldo

**Affiliations:** 1 Vascular Surgery, Fondazione Policlinico Universitario Campus Bio-Medico, Via Alvaro del Portillo, 200-00128 Roma, Italy; f.stilo@policlinicocampus.it (F.S.); n.montelione@policlinicocampus.it (N.M.); f.codispoti@policlinicocampus.it (F.A.C.); f.spinelli@policlinicocampus.it (F.S.); 2 Research Unit of Vascular Surgery, Department of Medicine, University Campus Bio-Medico di Roma, Via Alvaro del Portillo, 21-00128 Roma, Italy; a.ciolli@policlinicocampus.it (A.C.); j.paolini@policlinicocampus.it (J.P.); 3 Operative Research Unit of Anesthesia and Intensive Care, Fondazione Policlinico Universitario Campus Bio-Medico, Via Alvaro del Portillo, 200-00128 Roma, Italy; a.strumia@policlinicocampus.it (A.S.); e.tomaselli@policlinicocampus.it (E.T.); g.pascarella@policlinicocampus.it (G.P.); f.costa@policlinicocampus.it (F.C.); f.longo@policlinicocampus.it (F.L.); a.mattei@policlinicocampus.it (A.M.); l.schiavoni@policlinicocampus.it (L.S.); f.agro@policlinicocampus.it (F.E.A.); m.carassiti@policlinicocampus.it (M.C.); r.cataldo@policlinicocampus.it (R.C.); 4 Research Unit of Anaesthesia and Intensive Care, Department of Medicine, University Campus Bio-Medico di Roma, Via Alvaro del Portillo, 21-00128 Roma, Italy; alessandro.ruggiero@unicampus.it

**Keywords:** regional anesthesia, thoracic outlet syndrome, vascular surgery, thoracic surgery, pain, ultrasound

## Abstract

**Background:** Thoracic outlet syndrome (TOS) is an uncommon condition defined by the compression of neurovascular structures within the thoracic outlet. When conservative management strategies fail to alleviate symptoms, surgical decompression becomes necessary. The purpose of this study is to evaluate and compare the efficacy and safety of regional anesthesia (RA) using spontaneous breathing in contrast to general anesthesia (GA) for patients undergoing surgical intervention for TOS. **Methods:** We conducted a retrospective comparative study involving 68 patients who underwent trans-axillary first rib resection for TOS. The patient cohort was divided into two groups: 29 patients in the GA group and 39 patients in the RA group. The RA technique employed consisted of supraclavicular brachial plexus (SBP) and pectoral nerve (PECS II) blocks, accompanied by deep sedation. Key outcome measures such as pain scores, opioid consumption, and various perioperative parameters were systematically analyzed. **Results:** Postoperative pain levels recorded in the recovery room were significantly lower in the RA group, with a median numerical rating scale (NRS) score of zero compared to two in the GA group (*p* = 0.0443). Additionally, both intraoperative and postoperative opioid consumption showed a marked reduction in the RA group, with *p*-values of less than 0.001 and 0.0418, respectively. The RA approach was associated with shorter surgical durations (*p* = 0.0008), a decrease in the incidence of postoperative nausea and vomiting (PONV) (*p* = 0.0312), and a lower occurrence of intraoperative lung injuries (*p* < 0.0001). Furthermore, the length of hospital stay was significantly reduced for patients in the RA group. **Conclusions:** Although both groups reported low postoperative pain scores, the regional anesthesia approach exhibited distinct advantages in terms of opioid consumption, surgical duration, and overall perioperative outcomes. The utilization of SBP and PECS II blocks facilitated surgical procedures and mitigated complications, thereby positively influencing the postoperative recovery trajectory. Future prospective studies are essential to validate these findings further and to investigate long-term outcomes associated with the use of regional anesthesia in TOS surgery.

## 1. Introduction

Thoracic outlet syndrome (TOS) is a relatively uncommon condition, affecting less than 2% of the population [1]. It is characterized by the compression of the neurovascular bundle, which comprises the brachial plexus and the subclavian/axillary vessels, within the thoracic outlet. TOS is commonly categorized into four types: neurogenic TOS (NTOS), venous TOS (VTOS), arterial TOS (ATOS), and mixed TOS, based on the specific structures involved [2]. The clinical presentation of TOS can vary widely among individuals, encompassing a range of neurological symptoms such as upper extremity heaviness, fatigability, paresthesia, and pain, as well as vascular manifestations that may include swelling, venous distension, cyanosis, or thrombosis [3]. Although its treatment is predominantly conservative, surgical intervention is warranted when symptoms persist despite medical management.

Conservative treatment approaches typically include anti-inflammatory medications, weight loss, physical therapy, strengthening exercises, and botulinum toxin injections [2].

Various surgical techniques have been described for addressing TOS, all aimed at decompressing the thoracic outlet. The most common surgical interventions comprise brachial plexus decompression, neurolysis, and scalenectomy, with or without first rib resection or the removal of supernumerary ribs when present [2,3].

At our institution, we have been performing trans-axillary first rib resection for several years, establishing ourselves as a referral center at the national level [4]. This approach has proven to be safe and less invasive compared to conventional anterior thoracic techniques [4,5]. However, the surgical procedure is often associated with significant intraoperative and postoperative pain, considerable opioid consumption, and necessitates access to the pleural cavity.

Traditionally, general anesthesia has been the standard practice for thoracic interventions. Nevertheless, recent advancements in peripheral and fascial nerve blocks have demonstrated promising outcomes in thoracic surgery [6,7]. Regional anesthesia, particularly when employed with spontaneous breathing, has emerged as a viable alternative for anesthesia in select patients undergoing chest surgery, offering favorable results in terms of perioperative pain reduction and decreased opioid consumption [8,9].

The PECS II block, recently referred to as the pectoserratus block, has shown exceptional efficacy in thoracic and breast surgical procedures involving the axillary region, with evidence supporting its use for both analgesic and anesthetic purposes. Additionally, the supraclavicular brachial plexus (SBP) block is widely utilized in orthopedic surgeries for procedures concerning the arms, encompassing the T1 nerve root, which innervates the first rib.

This study aims to compare the effectiveness and safety of a combination of PECS II and SBP blocks with spontaneous breathing versus general anesthesia, specifically evaluating postoperative pain and opioid consumption in patients undergoing surgical decompression of the thoracic outlet with first rib resection using a minimally invasive trans-axillary approach.

## 2. Patients and Methods

This retrospective comparative study received approval from the Campus BioMedico Hospital Ethical Committee (protocol number PAR 24.21 ComEt CBM). All procedures were conducted in accordance with relevant laws and institutional guidelines.

We retrospectively enrolled a total of 68 consecutive patients who underwent trans-axillary first rib resection for thoracic outlet syndrome (TOS) between May 2020 and May 2023. Our previously published report describes the surgical technique employed for the decompression of the thoracic outlet [4,10].

Informed consent was obtained from all patients included in the study. The inclusion criteria consisted of an indication for trans-axillary first rib resection for TOS, patients aged over 16 years, and ASA physical status I-IV. Exclusion criteria encompassed allergies to local anesthetics, an infection at the puncture site, impaired cerebral function, patient refusal, and chronic opioid medication usage.

Patients were allocated into two groups based on the type of anesthetic management received:

- General Anesthesia (GA) Group: patients in this group underwent surgical intervention under general anesthesia without the use of regional blocks for analgesia;
- Regional Anesthesia (RA) Group: Patients in this group received a combination of supraclavicular brachial plexus (SBP) block and pectoral nerve (PECS II) block, with spontaneous breathing supported by deep sedation.

Both groups followed a standardized multimodal perioperative analgesia protocol that is routinely administered to all surgical patients at our institution. This protocol includes dexamethasone at a dosage of 0.1 mg/kg intravenously (IV) intraoperatively, ketorolac at 30 mg IV every 12 h, and paracetamol at 1 g IV every 8 h postoperatively. Additionally, morphine at a dosage of 2–3 mg IV was provided for patients with postoperative numerical rating scale (NRS) pain scores of 4 or higher. In our study, “low pain” was defined as NRS scores below 4, “moderate pain” as scores between 4 and 6, and “intense pain” as scores ranging from 7 to 10.

At the conclusion of the surgery, all patients were awakened in the operating room and subsequently transferred to the post anesthesia care unit (PACU) until they achieved an adequate Aldrete score before being moved to the surgical ward.

Patients in the GA group received anesthesia composed of sevoflurane at 2%, a continuous infusion of remifentanil at 0.1 mcg/kg/min (adjusted according to the anesthetist’s clinical judgment), fentanyl at 3–5 mcg/kg, rocuronium at 0.8–1 mg/kg, and propofol at 1% at a dose of 1–2 mg/kg for induction. The administration of additional boluses of fentanyl (1 mcg/kg) during the procedure was determined by the anesthetists based on hemodynamic parameter changes indicative of pain, such as a rapid increase in blood pressure and heart rate in the absence of fluid loading or vasopressor infusion. Bispectral index (BIS) monitoring was employed to assess the depth of anesthesia.

In the RA group, regional blocks were performed prior to the commencement of the surgical procedure. Patients subsequently received deep sedation with a continuous infusion of propofol at 2% in a target-controlled infusion (TCI) mode, achieving a target effect site concentration of 1 mcg/mL, alongside a continuous infusion of remifentanil at 0.1 mcg/kg/min (also adjusted according to anesthetist judgment), with supplemental oxygen delivered through a facial mask throughout the procedure. BIS monitoring was utilized to ensure appropriate sedation depth, while capnography was implemented for respiratory function monitoring. The anesthetists also administered boluses of fentanyl if changes in hemodynamic parameters suggested pain or if patient movement indicated discomfort.

### 2.1. Blocks Execution

In the operating room, patients in the regional anesthesia group were placed in the supine position while closely monitoring vital signs, including peripheral oxygen saturation (SpO_2_), electrocardiogram (ECG), and non-invasive blood pressure (NIBP). To enhance patient comfort, 2 mg of intravenous midazolam was administered.

For the supraclavicular brachial plexus (SBP) block, we utilized a high-frequency linear-array transducer (M-Turbo, Fujifilm-Sonosite, Bothell, WA, USA) to visualize the supraclavicular region. We identified the brachial plexus and the subclavian artery, both situated over the first rib.

A 100 mm needle (Stimuplex Ultra 360, B. Braun, Melsungen, Germany) was then inserted using an in-plane anteromedial approach, targeting the “corner pocket,” which is the angle formed between the artery and the rib. Following this, 10 mL of 2% mepivacaine was injected, and the distribution of the local anesthetic was observed around the brachial plexus. In cases of supernumerary ribs, due to altered anatomy, the needle was positioned lateral to the subclavian artery and beneath the brachial plexus.

**Figure 1:**
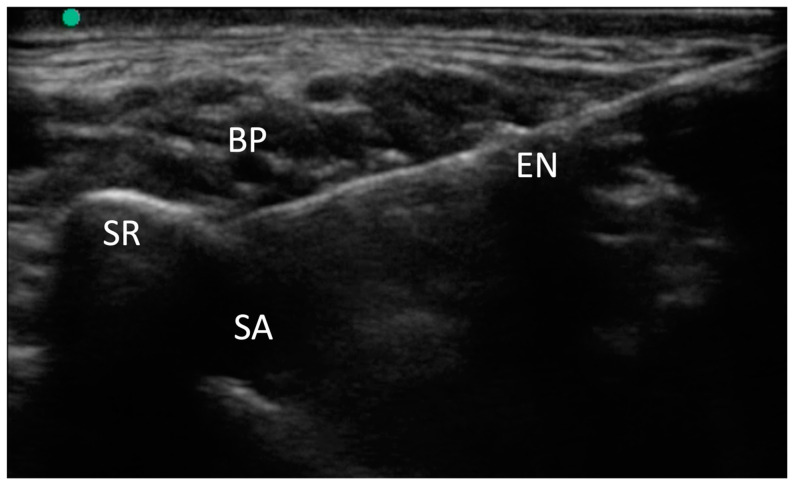
Supraclavicular brachial plexus block. The presence of a supernumerary rib alters the normal anatomy and sono-anatomy, thereby making a medial-to-lateral approach easier. BP: brachial plexus; SR: supernumerary rib; SA: subclavian artery; EN: echogenic needle.

This medial-to-lateral needle approach facilitated access to the supernumerary rib, as illustrated in Figure 1.

The PECS II block was performed using the same ultrasound linear transducer, positioned between the deltoid and pectoralis major muscles to identify the pectoralis major, the pectoralis minor, and serratus anterior muscles over the third rib. The 100 mm needle was inserted to target two distinct planes: the first between the pectoralis major and pectoralis minor muscles, where 10 mL of 0.375% ropivacaine was injected; the second between the pectoralis minor and serratus anterior muscles, where an additional 15 mL of 0.375% ropivacaine was administered, resulting in a total of 187.5 mg of ropivacaine.

The success of the blocks was assessed using the pinprick test to evaluate sensory loss in the axillary and ulnar regions. Once a sufficient level of sensory loss was achieved, sedation was given, and the surgical procedure commenced.

### 2.2. Data Collected

The primary outcome of the study was the assessment of postoperative pain levels upon admission to the recovery room.

Secondary outcomes included pain scores recorded during the first 24 h at various time points (6, 12, and 24 h), opioid consumption, surgical duration, the length of hospital stay, an incidence of postoperative nausea and vomiting (PONV), and the occurrence of postoperative complications (e.g., pneumothorax, hemothorax, and lung injury).

Data collected encompassed anthropometric measurement; the presence of diabetes mellitus; the side of surgery; the existence of a supernumerary rib; the type of TOS (neurogenic, venous, arterial, or mixed); the need for reintervention; postoperative pain at PACU admission and at 6, 12, and 24 h on a 0–10 NRS, reflecting the maximum pain experienced during those intervals; surgical length defined as the time from the first incision to the closure of the surgical wound; any conversions to general anesthesia within the RA group; adverse effects related to regional anesthesia; intraoperative opioid consumption (fentanyl and remifentanil); postoperative opioid consumption (total morphine usage in the first 24 h post-surgery); an incidence of PONV; postoperative complications (e.g., pneumothorax, hemothorax, and lung injury); and the length of stay (LOS) in the hospital.

All data were meticulously collected by anesthesiologists, vascular surgeons, or specialized nurses trained in the care of vascular and thoracic patients.

### 2.3. Statistical Analysis

To determine the appropriate sample size, we concentrated on our primary hypothesis which stated that perioperative analgesia is enhanced with regional anesthesia compared to general anesthesia alone in the recovery room. We retrospectively evaluated median numerical rating scale (NRS) pain scores from 15 patients who underwent trans-axillary first rib resection under general anesthesia, yielding an NRS score of 3 ± 1.7. Based on our calculations, at least 44 patients were needed to achieve an 80% probability of detecting, at a significance level of 5%, a reduction of at least 1.5 points in the NRS pain score, which is considered clinically significant [11].

Statistical analyses and graphical presentations were conducted using GraphPad Prism 8 (GraphPad Software Inc., San Diego, CA, USA) and Microsoft Excel. Mean values ± standard deviations (SD) were used to describe quantitative and continuous variables, while medians with interquartile ranges (IQRs) were applied for discrete variables. Qualitative variables were reported as counts and corresponding distribution percentages. The Shapiro–Wilk normality test was utilized to evaluate the parametric distribution of numerical variables. Differences between groups for parametric continuous variables were assessed using Student’s *t*-test, while the Wilcoxon-Mann–Whitney U test was applied when appropriate. Categorical variables were compared using the Pearson chi-square test. The threshold for statistical significance was established at *p*-values < 0.05.

## 3. Results

A total of 68 consecutive patients were enrolled in the study; among them, 29 underwent thoracic outlet syndrome (TOS) surgery under general anesthesia (GA), while 39 received regional anesthesia (RA) combined with deep sedation. Baseline characteristics did not differ significantly between the two groups, as shown in Table 1.

None of the patients in the RA group required conversion to general anesthesia, and no adverse effects associated with regional anesthesia were recorded.

**Figure 2:**
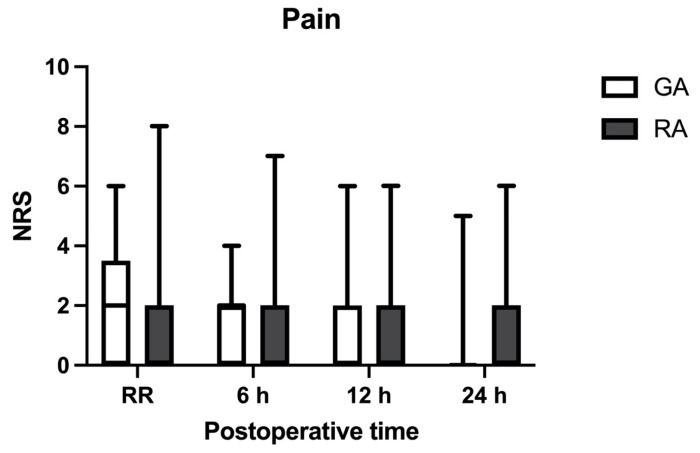
Postoperative pain scores. The box plot shows postoperative pain scores in the GA and RA groups. Data include pain reported at four different postoperative time points (RR, 6, 12, and 24 h). A 0–10 numeric rating scale (NRS) has been used to express pain, with 0 equal to no pain and 10 equals the worst imaginable pain. Values are expressed as the median (horizontal bars) with 25th–75th (box) as the range of minimum to maximum value (whiskers). RR = recovery room; GA = general anesthesia; RA = regional anesthesia.

Median postoperative pain levels in both groups remained low, with statistically significant differences observed only upon admission to the recovery room (RR). Specifically, patients in the GA group recorded a median NRS score of two (IQR 0–3), while those in the RA group reported a median score of zero (IQR 0–1), resulting in a *p*-value of 0.0443. At 6 h post-surgery, these differences lost statistical significance (*p* = 0.125), with median scores of two (IQR 0–2) in the GA group and zero (IQR 0–2) in the RA group. At the 12 h mark, median NRS values remained unchanged, with both groups showing a median score of zero (IQR 0–2) (*p* = 0.9603). Finally, at 24 h, the median NRS score for the GA group was zero (IQR 0–0) compared to zero (IQR 0–1) in the RA group, indicating no statistical differences (*p* = 0.4861). The pain trend is illustrated in Figure 2.

Conversely, intraoperative opioid consumption exhibited significant differences between the two groups, with reductions observed for both fentanyl (*p* < 0.001) and remifentanil (*p* = 0.0418), favoring the RA group. Similarly, postoperative morphine consumption demonstrated significant differences, again favoring the RA group. Data regarding opioid consumption are presented in Table 2.

Additional significant differences were noted in surgical duration (*p* = 0.0008), the length of stay (LOS) in the hospital (*p* = 0.0026), an incidence of postoperative nausea and vomiting (PONV) (*p* = 0.0312), and instances of intraoperative lung injury, which necessitated the placement of a hemopatch on the lung parenchyma in all cases (*p* < 0.0001). Secondary outcomes are summarized in Table 3.

## 4. Discussion

Thoracic outlet syndrome (TOS) is a rare condition that can manifest with a diverse array of symptoms, often resulting in delayed diagnosis. The optimal management of TOS remains a topic of debate. Typically, the first line of treatment is conservative, involving anti-inflammatory medications, weight loss, physical therapy, strengthening exercises, and botulinum toxin injections. This conservative approach appears to alleviate symptoms, particularly in patients with neurogenic TOS [2].

However, debilitating neurological and vascular symptoms associated with TOS frequently necessitate surgical intervention when less invasive treatments prove ineffective [12]. Various techniques have been described for the surgical decompression of the thoracic outlet, including supraclavicular, trans-axillary, and combined approaches. Common surgical procedures encompass brachial plexus decompression, neurolysis, and scalenectomy, which may be performed with or without first rib resection or removal of any supernumerary ribs present [2,3]. Notably, scalenectomy alone has been associated with a higher recurrence rate of the disease [13]. At our institution, we have been performing trans-axillary first rib resection for several years, establishing ourselves as a reference center in the country [4]. Despite the efficacy of this method, the procedure is not devoid of intraoperative and postoperative pain, contributing to significant perioperative opioid consumption [14]. Traditionally, as with other thoracic procedures, general anesthesia has been the standard practice for TOS surgery. However, advances in regional anesthesia have enabled the performance of various thoracic procedures in an “awake” state or with mild to deep sedation [15].

In addition to the PECS II block, other regional anesthesia techniques are gaining consideration for thoracic surgeries [16]. For instance, the serratus plane block (SPB) targets the serratus anterior muscle and intercostal nerves, providing effective analgesia for rib fractures, thoracic incisions, and chest wall muscle dissections. This technique is particularly valued for its simplicity and lower incidence of complications compared to methods such as the paravertebral block, which carries a higher risk of pneumothorax.

The erector spinae plane (ESP) block is another technique that has demonstrated promise in thoracic surgery. This block targets the erector spinae muscles and the dorsal rami of the spinal nerves, offering analgesia to the chest wall, including the paravertebral space and potentially the sympathetic nerves. The ESP block is becoming increasingly popular due to its ease of application, although its analgesic efficacy may be less predictable compared to other regional techniques. Other methods, such as the paravertebral block, remain frequently utilized in thoracic procedures, providing dense analgesia for the chest wall but posing a higher risk of complications, including pneumothorax and nerve damage. The epidural block is another viable option that offers excellent pain relief, particularly in extensive thoracic surgeries; however, it is associated with an increased risk of hypotension and motor block. Each of these techniques presents distinct advantages and limitations, and their application in TOS surgery is influenced by factors such as the patient’s condition, the surgical approach, and the anesthetic expertise available.

In this study, we aimed to compare general anesthesia (GA) with regional anesthesia (RA) using spontaneous breathing in conjunction with PECS II and SBP blocks for trans-axillary surgery addressing TOS, focusing on postoperative pain, opioid consumption, and various perioperative outcomes.

The PECS II block was utilized to anesthetize the following structures [17]: the pectoral nerves, the clavipectoral fascia, the intercostobrachial nerve, the lateral cutaneous branches of the third and fourth intercostal nerves, and the thoracodorsal nerve, thereby providing coverage for the skin incision and all surgical maneuvers involving the muscles.

The SBP block was administered to anesthetize the nerve plexus bundles near the first rib, mitigating pain and reflex responses during rib resection. Furthermore, considering that the primary innervation of the first rib arises from the T1 root, an area not consistently covered by more proximal brachial plexus block techniques, the SBP block was specifically selected. Additionally, this injection serves as a form of local infiltration for the first rib.

It is essential to recognize that the SBP block results in a temporary loss of motor function in the arm. In our study, mepivacaine was utilized for the SBP, providing the necessary anesthetic effect during surgery due to its short-acting properties, while its rapid half-life facilitated a swift recovery of arm function. Conversely, ropivacaine, used in the PECS II block, effectively managed postoperative pain due to its longer duration of action.

To the best of our knowledge, this investigation represents the first examination of TOS surgery employing regional anesthesia (RA) exclusively, without the adjunct of general anesthesia (GA). Nevertheless, several studies have previously explored the outcomes of regional blocks in combination with GA for this surgical procedure [14,18,19,20,21,22,23,24].

Pain levels following thoracic procedures are typically elevated [25]. In our investigation, however, postoperative pain demonstrated a statistically significant difference only in the recovery room, favoring the RA group. Pain scores subsequently converged in both groups at all analyzed time points. Notably, the median NRS scores remained consistently low across both groups, indicating an effective control of postoperative pain in all patients. Henshaw et al. also assessed the impact of the PECS II block in trans-axillary TOS procedures and reported no significant differences in pain levels between patients who received the block and those who did not [18]. Similarly, van den Broek and colleagues found no notable differences in their randomized trial [23]. In contrast, Goeteyn et al. observed that the addition of either the PECS II block or the PECS I block (administered between the pectoral muscles) combined with the erector spinae block significantly reduced postoperative pain and opioid consumption compared to patients who did not receive regional anesthesia [24]. Patel and colleagues also noted differences in pain management outcomes between patients treated with regional blocks and those who did not receive such interventions; however, the technique employed was distinct (the paravertebral block) [14]. Importantly, it should be emphasized that all patients enrolled in these studies received general anesthesia. Furthermore, the median or mean NRS scores documented in these investigations were generally higher than those observed in our study. This discrepancy may be attributed to variations in anesthesia techniques, surgical approaches, multimodal analgesia strategies, as well as differences in patient demographics (e.g., age, comorbidities, or pain sensitivity), postoperative care practices, the complexity or specific techniques of surgery, the efficacy of regional anesthesia blocks, cultural or reporting biases, and the timing and duration of pain assessments.

The observed similarity in pain levels between the two groups in our study may be elucidated by the significant differences noted in opioid consumption. Specifically, intraoperative consumption of fentanyl and remifentanil was markedly lower in the RA group, a trend that extended to postoperative morphine usage. In fact, 55% of patients in the GA group required postoperative morphine, whereas only 18% in the RA group did. Additionally, the median morphine consumption was statistically different between the two groups, again favoring the RA group. These findings align with the study conducted by Goeteyn et al. [24], while contrasting with Henshaw et al., where PECS II blocks did not demonstrate a reduction in opioid consumption [18]. It is important to reiterate that both groups in the studies by Goeteyn and Henshaw received GA. Although opioid utilization was lower in the RA group, it is crucial to recognize that multimodal analgesia was employed in both groups, potentially influencing the postoperative opioid consumption observed. The administration of non-opioid analgesics, such as acetaminophen and ketorolac, may have contributed to the decreased need for opioids in both groups following surgery. Opioid use further reflects the higher incidence of postoperative nausea and vomiting (PONV) in the GA group (31% vs. 10.3%; *p* = 0.0312).

**Figure 3:**
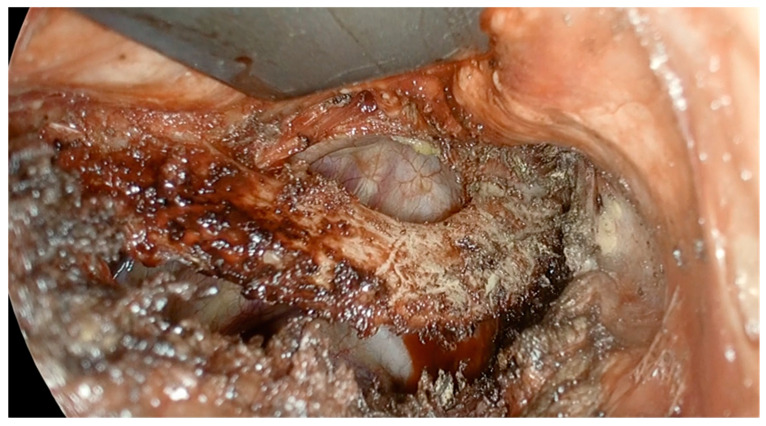
Intraoperative view of the first rib dissection. Of note is the large opening of the pleura which results in the collapse of the lung following an intentional pneumothorax.

The application of regional anesthesia has shown promising advantages not only from an anesthetic standpoint but also in terms of intraoperative surgical management. During the surgical dissection of the rib, the pleura is intentionally opened, and thanks to the spontaneous breathing facilitated by the RA, the lung collapses due to the creation of a controlled pneumothorax (Figure 3).

The absence of lung ventilation during the surgical procedure may have facilitated certain surgical maneuvers and potentially reduced both the duration of the operation and the risk of lung injuries. Indeed, surgical time was significantly shorter in the regional anesthesia (RA) group (123.27 ± 74.11 min in the general anesthesia (GA) group vs. 78.71 ± 24.86 min in the RA group; *p* = 0.0008). Furthermore, the intentional collapse of the lung during rib dissection might have likely enhanced safety for the surgeon, significantly decreasing the incidence of lung injuries that necessitated the application of an intraoperative hemopatch (GA 41% vs. 0% in the RA group). Achieving the same level of safety under GA would typically require selective bronchial intubation, a maneuver that is both more complex and carries greater risks compared to standard intubation techniques.

However, the induced pneumothorax and the potential risk of phrenic nerve impairment associated with the supraclavicular brachial plexus (SBP) block render this technique unsuitable for patients with pre-existing respiratory conditions. It is important to note that most patients with TOS undergoing surgical resection of the first rib in our series were relatively young (29–34 years), resulting in a low incidence of pulmonary comorbidities. Additionally, the intentional pneumothorax necessitated the placement of a chest drain in all GA patients, with complete resolution expected within 24 to 48 h.

Additionally, a contingency plan was established in all cases to address any airway complications that might arise during surgery. Despite these considerations, intraoperative hemodynamic parameters and oxygen saturation levels remained stable across all patients, and the surgeries were successful, with no conversions to GA in the RA group.

Moreover, the length of hospital stay was significantly shorter for the RA group compared to the GA group.

While this study offers valuable insights and establishes the feasibility of performing TOS surgery under regional anesthesia, several limitations must be acknowledged. Firstly, the retrospective nature of this study introduces inherent biases, such as selection bias and recall bias, which may affect the generalizability and internal validity of the findings. The relatively small sample size restricts the ability to draw broad conclusions and may not fully represent the diverse TOS patient population. A larger cohort would be necessary to confirm the results and potentially yield more robust findings.

Furthermore, other critical parameters, including intraoperative and postoperative arterial pressure, oxygen saturation, heart rate, urine output, and additional hemodynamic variables, were not documented. These factors could have influenced patient outcomes and provided further insights into the safety and effectiveness of regional anesthesia in TOS surgery. The absence of long-term follow-up constitutes another significant limitation, as chronic thoracic pain and other late complications were not assessed. Consequently, the durability of pain relief and the long-term effectiveness of regional anesthesia in managing recovery from TOS surgery remain uncertain. Additionally, no data on patient satisfaction were collected, leaving an important aspect of patient-reported outcomes unaddressed.

## 5. Conclusions

In conclusion, this study demonstrated the feasibility of trans-axillary surgery for TOS under regional anesthesia, specifically employing a combination of SBP and PECS II blocks with spontaneous breathing. Although the findings did not indicate an association between this approach and improved postoperative pain control, they did reveal reduced opioid consumption and other favorable outcomes, such as shorter surgical durations and hospital stays. Future prospective studies and randomized controlled trials are warranted to further validate the efficacy and safety of this regional anesthesia technique within the broader context of thoracic outlet syndrome surgery.

## Figures and Tables

**Table 1:** Basal characteristics.

	GA (n = 29)	RA (n = 39)	*p* Value
Age (yrs)	34.55 ± 9.61	29.92 ± 9.75	0.0539
Sex (M/F)	7/22	18/21	0.0626
BMI (kg/m^2^)	22.49 ± 2.38	28.5 ± 2.05	0.8377
ASA score, n (%)			
- I	8 (27.6%)	12 (30.7%)	
- II	21 (72.4%)	27 (69.3%)	
Smoke, n (%)	11 (38%)	14 (36%)	0.8634
Side, n (%)			0.462
- Dx	13 (45%)	21 (72.4%)	
- Sin	16 (55)	18 (27.6%)	
Supernumerary rib, n (%)	6 (20.7%)	8 (27.6%)	0.9858
Type of TOS, n (%)			
- VTOS	7 (24.1%)	14 (35.9%)	
- ATOS	2 (6.9%)	5 (12.8%)	
- NTOS	12 (41.4%)	9 (23%)	
- Mixed	8 (27.6%)	11 (28.3%)	
Reintervention, n (%)	4 (13.8%)	6 (15.4%)	0.8546

Values are reported as number (percentage) of subjects and mean ± standard deviation (SD). GA: general anesthesia; RA: regional anesthesia; BMI: body mass Index; VTOS: venous thoracic outlet syndrome; ATOS: arterial thoracic outlet syndrome; NTOS: nervous thoracic outlet syndrome.

**Table 2:** Opiate consumption.

	GA (n = 29)	RA (n = 39)	*p* Value
Intraoperative fentanyl, mcg	312.07 ± 92.24	96.15 ± 62.18	<0.0001
Intraoperative remifentanil, mcg	390.57 ± 390.71	73.13 ± 132.75	0.0418
Postoperative morphine, n (%)	16 (55%)	7 (18%)	0.0013
Total postoperative morphine, mg	3 (0–20)	0 (0–0)	0.0003

Values are reported as number (percentage) of subjects, mean ± standard deviation (SD), and median with interquartile range (IQR). GA: general anesthesia; RA: regional anesthesia.

**Table 3:** Secondary outcomes.

	GA (n = 29)	RA (n = 39)	*p* Value
Surgical time, min	123.27 ± 74.11	78.71 ± 24.86	0.0008
Lung injury, n (%)	12 (41%)	0 (0%)	<0.0001
LOS in hospital, days	4.48 ± 2.02	3.18 ± 1.41	0.0026
PONV, n (%)	9 (31%)	4 (10.3%)	0.0312
Hemothorax, n (%)	1 (3.4%)	2 (5.1%)	0.7387

Values are reported as number (percentage) of subjects and mean ± standard deviation (SD). GA: general anesthesia; RA: regional anesthesia; LOS: length of stay; PONV: postoperative nausea and vomiting.

## Data Availability

The raw data supporting the conclusions of this article will be made available by the authors on request.

## Abbreviations

TOS	thoracic outlet syndrome
PECS II	pectoral nerves block II
SBP	supraclavicular brachial plexus
ASA	American Society of Anesthesiology
PACU	post anesthesia care unit
GA	general anesthesia
RA	regional anesthesia
PONV	postoperative nausea and vomit
LOS	length of stay
RR	recovery room
NIBP	non-invasive blood pressure
SD	standard deviation
IQR	interquartile range
